# Haga usted el diagnóstico. Segunda parte

**DOI:** 10.7705/biomedica.7219

**Published:** 2023-08-31

**Authors:** Yenny Ariza, Cristian Leonardo Cubides, Daniel Alejandro Cubillos, Carmen Lucía Roa, José Camilo Álvarez, Sonia Isabel Cuervo-Maldonado

**Affiliations:** 1 Grupo de Medicina Interna e Infectología, Instituto Nacional de Cancerología, Bogotá, D.C., Instituto Nacional de Cancerología, Bogotá, D.C., Colombia Instituto Nacional de Cancerología Bogotá D.C Colombia; 2 Grupo de Investigación en Enfermedades Infecciosas en Cáncer y Alteraciones Hematológicas, Colombia Grupo de Investigación en Enfermedades Infecciosas en Cáncer y Alteraciones Hematológicas Colombia; 3 Facultad de Medicina, Universidad Nacional de Colombia, Bogotá, D.C., Colombia Universidad Nacional de Colombia Universidad Nacional de Colombia Bogotá D.C Colombia

En la segunda biopsia de ganglio axilar, no se informaron hallazgos de neoplasia maligna. Se observaron numerosos granulomas de diferentes tamaños, algunos con extensa necrosis central rodeada por histiocitos y algunas células gigantes multinucleadas; en el material necrótico, se apreciaron estructuras redondeadas rosadas. La coloración para bacilos ácido-alcohol resistentes (Ziehl-Neelsen) fue negativa; las coloraciones para hongos y la de PAS fueron negativas. La coloración de Gomori fue positiva para numerosas estructuras micóticas redondas, algunas en “timón de barco”. Los hallazgos se interpretaron como la presencia de numerosas estructuras micóticas sugestivas de *Paracoccidioides* spp. (figura 1). Los cultivos para gérmenes comunes, micobacterias y hongos fueron negativos.

**Figura 1:**
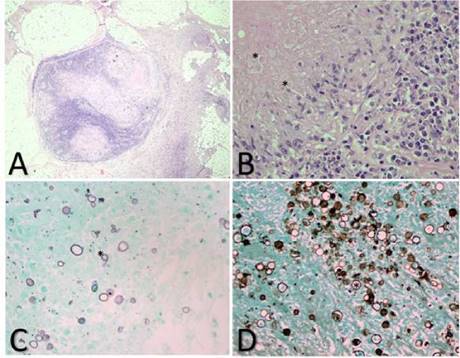
Biopsia de ganglio linfático izquierdo. A. Se observa ganglio linfático con numerosos granulomas de diferentes tamaños, algunos con extensa necrosis central rodeada por histiocitos y algunas células gigantes multinucleadas; en el material necrótico se aprecian estructuras redondeadas rosadas. B. Se observan levaduras esféricas de doble pared, de 30 a 60 μm de diámetro. Hematoxilina y eosina, 20X. C y D. Se aprecian numerosas estructuras micóticas redondas, alguna en "timón de barco". Gomori, 20X.

Se planteó el diagnóstico de paracoccidioidomicosis ganglionar. Se inicio tratamiento con deoxicolato de anfotericina B durante 14 días, y se observó mejoría de las adenopatías cervicales y axilares en su tamaño y características.

Se dio de alta al paciente, para continuar el tratamiento antimicrobiano por doce meses y con las alternativas terapéuticas disponibles, itraconazol versus trimetoprim-sulfametoxazol. Dada la condición social del paciente, se decidió formularle trimetoprim-sulfametoxazol y hacer control médico por consulta externa de infectología.

## Paracoccidioidomicosis ganglionar juvenil

### Resumen

La paracoccidioidomicosis es una enfermedad endémica del continente latinoamericano, específicamente en zonas tropicales, y afecta con mayor frecuencia y de manera crónica a individuos inmunocompetentes; sin embargo, existen formas agudas y de presentación diseminada. El agente patógeno responsable es *Paracoccidioides brasiliensis*, un hongo dimorfo que se transmite por inhalación y cuya progresión depende del control y la vulnerabilidad del sistema inmunológico.

En este caso clínico, se expone la historia de un hombre de 23 años procedente de Venezuela, sin antecedentes de importancia, que consultó por dolor lumbosacro crónico, múltiples linfadenopatías y hepatoesplenomegalia. Inicialmente, se sospechó una linfadenitis bacteriana o un posible linfoma no Hodgkin, por lo cual se remitió a un centro de referencia en cáncer.

Los estudios de extensión y la histopatología demostraron levaduras en disposición de “timón de barco”, lo cual confirmó el diagnóstico de paracoccidioidomicosis. Finalmente, se observó mejoría de las lesiones con el tratamiento antifúngico dirigido y se continuó con tratamiento oral ambulatorio.

Este caso permite demostrar la complejidad del diagnóstico de la paracoccidioidomicosis por sus múltiples diagnósticos diferenciales y por las diferentes manifestaciones que puede presentar según el huésped. El objetivo de presentar este caso fue recordar al lector que las micosis endémicas en población migrante son un diagnóstico diferencial que se debe considerar en el abordaje de las linfadenitis periféricas.

Palabras clave: paracoccidioidomicosis; linfadenitis; micosis; adulto joven; inmunocompetencia; emigrantes e inmigrantes.

## Juvenile node paracoccidioidomicosys

### Abstract

Paracocciodiodomycosis is an endemic disease of the Latin American continent, specifically in tropical areas, and it most frequently affects chronically immunocompetent individuals; however, there are acute and disseminated forms. The responsible pathogen is *Paracoccidioides brasiliensis*, a dimorphic fungus transmited by inhalation and whose progression depends on the control and susceptibility of the immune system.

The following clinical case presents the history of a 23-year-old man from Venezuela, with no significant medical history, who consulted for chronic lumbosacral pain, multiple lymphadenopathies, and hepatosplenomegaly. Bacterial lymphadenitis or a possible nonHodgkin lymphoma were initially suspected. He was referred to a cancer center, where extension studies and histopathology showed the presence of yeast in a “captain’s wheel” formation, confirming the diagnosis of paracoccidioidomycosis. Finally, a targeted antifungal treatment was performed, observing improvement of the lesions and he continued as an outpatient with oral treatment.

This case allows to demonstrate the complexity of the diagnosis of paracoccidioidomycosis due to its multiple differential diagnoses and the different manifestations that it can present depending on the host. The purpose of reporting this was to remind the reader that endemic mycoses in the migrant population are a differential diagnosis that should be considered in the approach to peripheral lymphadenitis.

Key words: Paracoccidioidomycosis; lymphadenitis; mycoses; young adult; immunocompetence; emigrants and immigrants.

## Introducción

La paracoccidioidomicosis es una micosis sistémica endémica en Latinoamérica, más frecuente en Brasil, Argentina, Colombia y Venezuela, causada por un hongo dimorfo, *Paracoccidioides brasiliensis*, el cual incluye cuatro grupos genético*s S1, PS2, PS3, PS4 y* una nueva especie, *Paracoccidioides lutzii*; esta última fue descrita en la región medio-occidental de Brasil, principalmente en el Estado de Mato Grosso, con datos clínicos y de laboratorio muy limitados que, comparados con los del perfil clínico- epidemiológico propio de *P. brasiliensis*, no muestran diferencias particulares ^1^.

Turissini *et al.*^2^, en un estudio de 65 aislamientos de *P. brasiliensis*, compararon los polimorfismos del ADN nuclear y mitocondrial y, con estos datos, establecieron una nueva clasificación del complejo *P. brasiliensis* compuesto por cuatro nuevas especies: *P. brasiliensis sensu stricto* (clados S1a y S1b), *P. americana* (PS2), *P. restrepiensis* (PS3) y *P. venezuelensis* (PS4). *Paracoccidioides brasiliensis sensu stricto* está ampliamente disperso en el sudeste de Brasil, Argentina y Paraguay; *P. americana* (PS2) ha sido reportado en el sureste de Brasil y raramente en Venezuela; *P. restrepiensis* (PS3) se encuentra predominantemente en Colombia y en otros países latinoamericanos, y *P. venezuelensis* (PS4) parece ser dominante en Venezuela ^3^.

El hongo está presente en la naturaleza como filamentos que contienen propágulos infecciosos llamados conidios. En áreas endémicas de Latinoamérica, la infección se adquiere por inhalación de los conidios presentes en suelos contaminados con el hongo, los cuales dan lugar a levaduras, que es la forma parasitaria en los tejidos del huésped ^4^. El principal factor de riesgo para adquirir la infección son las profesiones o actividades relacionadas con el manejo de suelos contaminados con el hongo, tales como actividades agrícolas, movimiento de tierras, preparación del suelo, jardinería y transporte de productos vegetales.

En las zonas endémicas, la infección se adquiere principalmente en las primeras dos décadas de la vida, pero las manifestaciones clínicas ocurren con mayor frecuencia en adultos entre los 30 y los 50 años, después de la reactivación del foco endógeno latente ^5^. Según el estado inmunológico del huésped, la infección se puede resolver espontáneamente o puede progresar en forma aguda, subaguda o crónica. La presentación linfática corresponde a la paracoccidioidomicosis ganglionar juvenil, la cual es más frecuente en personas jóvenes, como el caso que se presenta.

## Epidemiología

La epidemiología de la paracoccidioidomicosis está bien establecida. Brasil es uno de los muchos países con un gran número de pacientes y se sabe que esta enfermedad es particularmente endémica en el estado de Minas Gerais, hecho que habla de la amplia variación ambiental entre las diferentes áreas dentro de este estado ^6^. Restrepo *et al.* revisaron varios estudios sobre la ecología de *P. brasiliensis*; a pesar de algunas incertidumbres sobre su ecología específica, se sabe que la paracoccidioidomicosis florece en regiones de temperatura y alturas moderadas, clima húmedo con copiosas precipitaciones y zonas con abundantes recursos hídricos ^7^. Estas condiciones se cumplen en varias regiones con zonas de bosques tropicales y subtropicales, que son predominantes en el continente suramericano. En el contexto de la globalización, se espera que la paracoccidioidomicosis sea más frecuente en otros países como una enfermedad importada, lo que representa un nuevo problema de salud emergente para los países con frecuentes viajeros internacionales e inmigrantes.

El hábitat que permite el desarrollo de la forma infectante corresponde a áreas en un rango de temperatura entre los 10 y los 28 °C, con una precipitación entre 500 y 2.500 mm por año y una altitud de 47 a 1.300 msnm ^8^. Estas características las cumple el país de procedencia del paciente, Venezuela, el cual es el tercero más afectado por la paracoccidioidomicosis en Latinoamérica, a pesar de que se estima que existen un importante subdiagnóstico y una escasa notificación.

Primavera *et al.,* en un estudio de 745 casos registrados en Venezuela durante 65 años, entre 1954 y 2019, informan casos de paracoccidioidomicosis en varios estados, siendo el Estado de Miranda el que más casos aporta, seguido de Guárico, Aragua, Carabobo y Anzoátegui (1 a 3 casos); este último, era el lugar de procedencia del paciente ^8^. En las formas de presentación agudas o subagudas, se informa compromiso ganglionar hasta en el 82 % de los casos. Entre 1930 y 2012, la mediana de casos por año en Venezuela fue de 25,9 casos ^9^; la mortalidad varía entre el 6,1 y el 7,6 % ^10^.

Por otra parte, se encontró que el 32,48 % de los pacientes afectados con paracoccidioidomicosis eran trabajadores rurales, trabajadores de mantenimiento general o del área de la construcción ^7^.

En toda Colombia, se ha reportado una incidencia entre 0,5 y 2,2 por 100.000 habitantes y, en los seis municipios con mayor incidencia, una entre 0,8 y 3,1 por 100.000 habitantes. La mediana de casos por año en Colombia fue de 32,4 entre 1930 y 2012 ^9^.

De igual manera, Torrado *et al.* estudiaron 1.191 casos en un estudio retrospectivo entre 1949 y 1999. Nueve departamentos se clasificaron como endémicos, ya que informaron más de 32 casos por año; ocho corresponden a la región andina (Antioquia, Santander, Cundinamarca, Meta, Norte de Santander, Caldas, Boyacá y Tolima) y el otro es el departamento del Magdalena (Sierra Nevada de Santa Marta) ^11^. Dieciocho departamentos se catalogaron como regiones de baja endemicidad (2 a 27 casos) y, los 5 restantes, como no endémicos (1 caso). Entre los departamentos de baja endemicidad, se encuentra el de Casanare, sitio de procedencia del paciente.

Por otra parte, entre 1980 y 1998, y con base en el diagnóstico anual de casos, se encontraron ocho departamentos donde se había diagnosticado la enfermedad por periodos de 13 a 19 años, lo que permitió clasificarlos como endémicos. Además, 15 se consideraron de baja endemicidad, con base en un período de diagnóstico de 2 a 12 años. Los restantes 10 fueron clasificados como no endémicos, por haber informado la entidad solamente en un año. La mayor incidencia nacional, 2,4 pacientes por 1’000.000 habitantes, se obtuvo en 1980. Debido a que esta no es una enfermedad de reporte obligatorio, no se tienen datos precisos de la incidencia y la mortalidad ^12^^,^^13^.

Según Martínez y el mapa de endemicidad de la paracoccidioidomicosis en Latinoamérica, la zona central de Colombia y la zona centro-oriental de Venezuela son aquellas con mayor número de casos informados ^9^. El paciente es natural del estado de Anzoátegui, en el que se han informado pocos casos de la enfermedad; además, durante su estancia en Colombia, ha permanecido en un municipio con muy pocos casos. Según nuestro conocimiento, este es el tercer caso de paracoccidioidomicosis ganglionar juvenil informado en Colombia. ^11^^,^^14^^,^^15^.

Según la endemicidad de la paracoccidioidomicosis en Venezuela y en Colombia, y la forma de presentación de la enfermedad (ganglionar juvenil), se considera que el paciente adquirió la infección en Venezuela y la desarrolló en Colombia.

## Fisiopatología

La paracoccidioidomicosis es causada, en gran medida, por la composición antigénica y los mecanismos de virulencia del hongo; por ende, la reacción inmunológica del huésped juega un papel determinante, en el que las variantes genéticas facilitan la presentación o el control de la enfermedad ^16^.

El agente patógeno se transmite por inhalación. Por esto, la reacción inmunológica se inicia en el alvéolo pulmonar, en donde el hongo se fija a proteínas de la matriz extracelular e interactúa con las células epiteliales. Estas activan la reacción inmunológica innata, en la que los macrófagos alveolares son la principal defensa contra este agente patógeno, al fagocitar y eliminar las conidias y las levaduras, mediante mecanismos asociados con el metabolismo oxidativo. La reacción que generan es inespecífica y se acompaña de la formación de granulomas ^17^.

Posteriormente, la reacción inmunológica adaptativa juega un papel fundamental en el control del agente patógeno, el cual va a depender de la reacción de hipersensibilidad tardía contra el hongo y sus antígenos, ya que, por sus mecanismos de virulencia, este puede estimular la apoptosis de linfocitos y aumentar la expresión de citocinas antiinflamatorias y, con ello, de linfocitos reguladores.

En pacientes afectados por la enfermedad, se ha identificado una reacción de linfocitos Th2 (T helper 2) asociados con una reacción humoral. En pacientes con la infección, que habitan en zonas endémicas, se presentan reacciones y perfiles celulares asociados con linfocitos Th1 (T helper 1); estos conllevan una reacción mediada por células, especialmente macrófagos y células T citotóxicas, lo que demuestra una mejor reacción contra el hongo ^1^^,^^16^.

En individuos propensos que tengan una infección primaria, el curso clínico de la enfermedad puede tomar diferentes rutas. Una de ellas ocurre especialmente en niños, adolescentes y pacientes inmunocomprometidos, en quienes el sistema inmunológico es incapaz de controlar el agente patógeno y la infección se disemina. Esta se denomina una infección sistémica rápidamente progresiva o una forma aguda o subaguda. La otra forma de la enfermedad se presenta en adultos en quienes el control es parcial, y la diseminación del agente patógeno se produce de manera crónica por medio del sistema linfático y el venoso ^16^.

## Clasificación

De acuerdo con el Coloquio Internacional de 1986, la paracoccidioidomicosis se clasifica según las manifestaciones clínicas y la historia natural de la enfermedad, en las siguientes categorías: infección asintomática; enfermedad con manifestaciones clínicas; forma aguda en niños y subaguda en adolescentes; forma crónica en adultos (unifocal o multifocal), y forma residual ^18^.

## Formas clínicas

### Aguda o subaguda en niños y adolescentes

Esta forma clínica de la paracoccidioidomicosis afecta niños, adolescentes *y* adultos hasta los 35 años de edad, y corresponde del 3 al 5 % de todos los casos ^18^. Representa la evolución directa del foco primario pulmonar y puede progresar con un curso clínico agudo o subagudo de la fungemia.

Aunque todos los sistemas y órganos se pueden comprometer, el sistema reticuloendotelial es el más afectado. El cuadro clínico predominante se caracteriza por linfadenopatía generalizada (superficial y profunda) y hepatoesplenomegalia, por lo que esta presentación se puede confundir con enfermedad linfoproliferativa sistémica, como ocurrió con el caso que se presenta; según el grado de diseminación, se puede subtipificar como forma moderada o grave. Se pueden identificar varios cuadros clínicos, como: 1) síntomas gastrointestinales (diarrea, dolor abdominal), masas o ambos; 2) signos y síntomas de afectación osteoarticular, que simulan osteomielitis o artritis; 3) síndrome abdominal agudo. La afectación de mucosas y pulmón es infrecuente, aunque la enfermedad puede comenzar como una infección respiratoria o con lesiones cutáneas ^5^^,^^14^^,^^15^.

### Forma crónica focal o multifocal en adultos

Es la forma más común de la paracoccidioidomicosis (90 %) y afecta con más frecuencia a los hombres. La forma unifocal se caracteriza porque la evolución es crónica, con síntomas respiratorios e infiltrado retículo-nodular pulmonar, generalmente, con afectación de los dos tercios superiores. La forma multifocal incluye el compromiso extrapulmonar de piel, mucosa oral ^19^, faríngea, laríngea y de la encía (ápices dentales), por lo que los síntomas pueden incluir dolor asociado con la masticación, sialorrea y odinofagia. Otros órganos afectados incluyen glándulas suprarrenales, sistema nervioso central ^20^, ganglios linfáticos superficiales y profundos, sistema macrófago- monocito (hígado y bazo), sistema osteoarticular y epididimitis.

La paracoccidioidomicosis abdominal se observa en la forma subaguda, con una amplia variedad de manifestaciones clínicas, que van desde náuseas, vómitos, ascitis, ictericia, dolor abdominal variable, hepatoesplenomegalia, malabsorción, masa tumoral, obstrucción intestinal, peritonitis y mesenteritis, hasta abdomen agudo por perforación de víscera hueca. La ictericia suele aparecer en una etapa tardía y está asociada con una enfermedad más grave. Normalmente, es causada por compresión extrínseca del colédoco por ganglios linfáticos. Otras causas descritas son lesión granulomatosa intraluminal del colédoco (lesión intrínseca), lesión hepática y paracoccidioidomicosis pancreática ^21^.

## Diagnósticos diferenciales

El principal diagnóstico diferencial de la paracoccidioidomicosis es la tuberculosis pulmonar y están también micobacterias atípicas, sarcoidosis, histoplasmosis, neumonitis intersticial idiopática difusa, silicosis crónica, coccidioidomicosis, cromoblastomicosis, leishmaniasis cutánea y visceral, lepra, y cáncer cutáneo o laríngeo.

En el caso aquí presentado, el diagnóstico diferencial considerado en el primer lugar en donde consultó fue el de linfoma por causa de la linfadenitis cervical y axilar; sin embargo, en la segunda biopsia tomada del ganglio axilar, no se encontraron signos de malignidad. Por otra parte, y aunque la asociación de paracoccidioidomicosis con tuberculosis se describe hasta en el 30 % de los casos ^22^, el resultado de los cultivos del ganglio axilar fue negativo.

## Diagnóstico

El diagnóstico en el laboratorio está dirigido a la visualización del hongo en muestras clínicas o su aislamiento mediante cultivo; la cuantificación de anticuerpos o la detección de antígenos del hongo en el suero, pueden considerarse como criterios diagnósticos indirectos ^10^.

El examen directo tiene una sensibilidad entre el 85 y el 100 %, la cual depende de la muestra; así, en muestras de las vías respiratorias con técnicas como montaje húmedo en esputo, exudados o el lavado broncoalveolar, se revela el hongo en más del 85 % de los casos. En el examen micológico directo positivo se pueden observar levaduras grandes (5 a 15 pm) que tienen una pared celular gruesa y birrefringente, con brotes únicos o múltiples. Estos brotes múltiples tienen forma de “volante” o “Mickey Mouse” y se consideran hallazgos patognomónicos en el diagnóstico de paracoccidioidomicosis.

Para el estudio histopatológico de la biopsia, se recomienda la coloración de plata con metenamina de Gomori, en la que se puede observar la levadura en gemación múltiple (figura 3). Con la de hematoxilina y eosina, se evidencian granulomas con células multinucleadas gigantes, células epitelioides, mononucleares y neutrófilos (figura 3) ^23^.

El cultivo en agar Sabouraud dextrosa a temperatura de 37 °C confirma la infección activa. Sin embargo, el resultado tarda entre 20 y 30 días, es positivo únicamente en el 85 % de las muestras y tiene alto riesgo de contaminación, por lo cual deben hacerse múltiples cultivos de repetidas muestras y, además, en diferentes medios de cultivo ^24^.

Por último, otras técnicas diagnósticas inmunológicas son de utilidad para el seguimiento del paciente; entre estas, se encuentra la prueba de inmunodifusión en agar, que puede demostrar la presencia de anticuerpos en más del 90 % de los casos; además, está la prueba de fijación de antígeno, que puede presentar reacción cruzada con antígenos de *Histoplasma capsulatum.* También se usan otras técnicas como ELISA e inmunotransferencia (*immunoblotting*).

Los cambios taxonómicos impulsados por la secuenciación genómica completa de *Paracoccidioides*, han puesto de relieve la necesidad de reconocer las especies. Actualmente, las técnicas clásicas de laboratorio no tienen un poder discriminatorio significativo y, hasta la fecha, se desconoce la utilidad clínica de identificar la especie. Varios métodos basados en la reacción en cadena de la polimerasa (PCR) pueden detectar polimorfismos en el ADN e identificar las especies, entre los cuales se puede mencionar; PCR; polimorfismo de longitud de fragmentos de restricción (RFLP); reacción en cadena de la polimerasa cuantitativa en tiempo real (qPCR); hibridación fluorescente *in situ* (FISH), y amplificación isotérmica mediada por bucle (LAMP). Sin embargo, aún no se dispone de una prueba comercial ^23^.

## Secuelas y complicaciones

Las secuelas de la paracoccidioidomicosis dependen del compromiso orgánico inicial, el estado inmunológico del huésped, la edad, algunas comorbilidades y el tiempo de evolución de la enfermedad. Como en la mayoría de los casos se evidencia compromiso pulmonar, las secuelas que se observan con mayor frecuencia corresponden a fibrosis pulmonar secundaria, alteración en la arquitectura del pulmón y compromiso de la función pulmonar: en orden de frecuencia, se describen fibrosis (32 %), bulas (27 %), signos de hipertensión pulmonar, hipertrofia ventricular derecha y *cor pulmonale* secundario.

En cerca del 85 % de los casos, el compromiso de la glándula suprarrenal encontrado en la autopsia es similar al de la enfermedad de Addison y es potencialmente reversible si se administra el tratamiento antifúngico adecuado.

Cuando hay compromiso de mucosas (a diferencia de la tuberculosis, la cual usualmente no las compromete), las secuelas son pérdidas dentarias, alteraciones de la anatomía propia de la cavidad orofaríngea, el paladar, la lengua y los posibles tejidos circundantes en labios, en nariz o en ambos. También, se pueden comprometer las mucosas de la faringe, la laringe y del tubo digestivo, en el cual se evidencia mayor compromiso en el colon y el íleon terminal ^25^.

Si hay compromiso en la piel, pueden encontrarse secuelas cicatriciales, y retracciones de la piel y el tejido celular subcutáneo. En ocasiones, pueden encontrarse placas infiltrativas similares a las lesiones producidas por sarcoidosis y, en menor proporción, se pueden evidenciar granulomas residuales.

El compromiso del sistema nervioso central es raro (aproximadamente en el 9 % de los casos), pudiéndose manifestar como un efecto seudotumoral o de masa expansiva, con afectación de los hemisferios cerebrales (el 69 % de las lesiones se ubican allí) o como síndrome de compresión medular o espondilodiscitis. Sin tratamiento adecuado, se puede presentar paraplejia, incontinencia urinaria y estados de postración ^26^.

## Tratamiento

El itraconazol es el medicamento usado para tratar las formas leves a moderadas de la infección, el cual ha demostrado ser superior a otros antimicrobianos como el cotrimoxazol, en cuanto a tasa de éxito y menor tiempo de cura serológica. El itraconazol tiene una tasa de éxito del 91 % usado durante seis meses; sin embargo, se recomienda que la duración sea de 9 a 12 meses y, para el caso del cotrimoxazol, de 18 a 24 meses ^23^. En pacientes inmunocomprometidos y presentaciones graves y diseminadas, se recomienda el deoxicolato de anfotericina B durante 2 a 4 semanas como terapia de inducción. Después de ello, se recomienda un tratamiento de mantenimiento con antifúngicos de la familia de los azoles. Para el tratamiento de la forma aguda o subaguda, se puede usar el cotrimoxazol por su tolerabilidad y su presentación tanto oral como intravenosa ^27^.

## Criterios de curación

Una vez la enfermedad es diagnosticada, el tratamiento requiere un seguimiento periódico, ya que este no erradica al hongo, sino que reduce la carga fúngica en el individuo y permite la recuperación con la reacción inmunológica. Dicho seguimiento se puede hacer de manera ambulatoria.

Teniendo en cuenta esto, la duración del tratamiento también se puede ajustar según los criterios de curación que son clínicos, micológicos, radiológicos e inmunológicos ^23^.

En cuanto a los criterios clínicos, se requiere la ausencia o regresión de los signos y síntomas de la enfermedad, los cuales indican una respuesta favorable al tratamiento. Entre estos criterios, se pueden encontrar la mejoría de la lesión en piel, la involución de las adenomegalias y la recuperación del peso corporal. Cabe mencionar que es frecuente la persistencia de síntomas residuales o secuelas asociadas con fibrosis y cicatrización en órganos como los pulmones, el sistema linfático y las glándulas suprarrenales, y deben diferenciarse de la presentación clínica de la infección activa.

Los criterios micológicos se refieren a que el examen microscópico directo se torne negativo en muestras clínicas. Este criterio puede ser verificado, especialmente en secreciones respiratorias.

Para cumplir con los criterios radiológicos, se hace un seguimiento semestral de las imágenes torácicas, buscando una estabilización del patrón radiológico, ya que se da una transición de patrones nodulares, micronodulares o, incluso, lesiones cavitadas que se vuelven lineales con el tiempo, lo cual indica cicatrización y fibrosis ^28^.

En cuanto a los criterios inmunológicos, si es posible, se hace seguimiento serológico cada seis meses. La técnica más frecuentemente usada es la reacción de inmunodifusión en agar, cuyos títulos de anticuerpos se vuelven negativos o se estabilizan en valores bajos con el tratamiento. Otra técnica utilizada es la contrainmunoelectroforesis.

Sin embargo, no se aplica el término de cura definitiva en pacientes con paracoccidioidomicosis, porque *P. brasiliensis* no es erradicado del organismo. Por ello, después del tratamiento y el cumplimiento de estos criterios, los pacientes deben seguir en controles clínicos y serológicos, por lo menos, un año después ^23^.

## Asociación entre paracoccidioidomicosis e inmunosupresión

La asociación potencial entre la paracoccidioidomicosis y el cáncer -la cual fue descrita por primera vez en 1933, debido a que el hongo tiene afinidad por comprometer el pulmón- es de particular interés. Aunque no se ha comprobado que la incidencia de cáncer en pacientes con paracoccidioidomicosis sea mayor que en la población general, la enfermedad se ha asociado con el cáncer de pulmón ^29^, y con el linfoma Hodgkin y no Hodgkin ^30^.

De otra parte, la paracoccidioidomicosis abdominal con ictericia más otros síntomas inespecíficos, se puede confundir con el colangiocarcinoma. Por esto, ante la presencia de ictericia obstructiva y sospecha de colangiocarcinoma, en Brasil y otras áreas endémicas de América Latina, se debe considerar la paracoccidioidomicosis como un diagnóstico diferencial ^21^.

## Conclusión

La paracoccidioidomicosis es una enfermedad fúngica endémica en Latinoamérica, cuyas formas de presentación clínica son diversas. Las formas agudas y diseminadas, como la expuesta en el presente caso, son un reto diagnóstico por su baja incidencia y su cuadro clínico, que incluye múltiples diagnósticos diferenciales como neoplasias hematológicas malignas, por su compromiso linfático y diseminado. Por ende, siempre debe considerarse la forma subaguda o aguda de la paracoccidioidomicosis en pacientes provenientes de zonas endémicas, especialmente, si realizan labores agrícolas en áreas rurales y presentan síntomas asociados con el sistema linfático, el respiratorio o el tegumentario.

El tratamiento es la administración de itraconazol o cotrimoxazol en casos leves a moderados. Si la infección es grave, se considera la administración de deoxicolato de anfotericina B. El tratamiento es prolongado, ya que la erradicación del hongo es imposible de lograr y se requiere un seguimiento médico continuo del paciente.
